# Fewer poor joint awareness results with cementless TKA compared with the same-design cemented implant

**DOI:** 10.1007/s00402-026-06531-w

**Published:** 2026-10-09

**Authors:** Luca De Berardinis, Filippo Leggieri, Paolo Salari, Giuseppe Polizzotti, Andrea Baldini

**Affiliations:** 1 Orthopaedic and Trauma Unit, IRCCS Ospedale San Raffaele, Milan, Italy; 2 Institute of Biomechanics, Paracelsus Medical University (PMU), Salzburg, Austria; 3 University of Florence, Florence, Italy; 4 Orthopaedic Unit, Istituto Fiorentino di Cura e Assistenza, Firenze, Italy; 5 Orthopaedic Unit, Istituto Clinico San Rocco di Ome, Brescia, Italy

**Keywords:** Total knee arthroplasty, Cemented, Cementless, 3D printing, Full-titanium

## Abstract

**Background:**

Comparisons of cemented and cementless TKA have focused on mean outcomes and generally reported equivalence. We hypothesized that a 3D-printed full-titanium cementless TKA and the same-design cemented implant would not differ significantly in average joint awareness but would differ in the proportion of patients with the poorest results, and we compared the two both at the mean and across the outcome distribution.

**Methods:**

Prospective two-cohort study of 109 primary unilateral TKAs (54 cementless, 55 cemented) for end-stage knee osteoarthritis, with complete 24-month follow-up. Exclusion criteria were valgus deformity and bilateral surgery. The primary outcome was the FJS-12, analysed both at the mean (multivariable linear regression, with propensity score overlap weighting as sensitivity analysis) and across its distribution (quantile regression). Secondary outcomes were PASS achievement (FJS-12 ≥ 33.3), OKS, and time to functional satisfaction.

**Results:**

The cementless group was younger (*P* < 0.001) with more men (*P* = 0.008). Mean FJS-12 was not different between groups after adjustment (β = 7.4, 95% CI − 1.8 to 16.6, *P* = 0.115), although the confidence interval did not exclude a difference exceeding the minimal clinically important difference; this finding was reproduced by overlap weighting. On quantile regression, cementless patients scored significantly higher at the 10th and 25th percentiles (adjusted *P* = 0.028 and 0.021) but not at the median or above. A greater proportion of cementless patients achieved the PASS (96.3% vs. 76.4%; absolute difference 19.9%, 95% CI 7.1 to 32.9; number needed to treat 5), which persisted after adjustment (*P* = 0.005). No significant differences were observed in OKS (*P* = 0.91) or time to functional satisfaction (adjusted *P* = 0.39). No revisions or loosening occurred.

**Conclusion:**

The 3D-printed full-titanium cementless TKA did not produce a significantly higher mean FJS-12 than the same-design cemented device at 24 months, but it significantly reduced the proportion of patients with poor joint awareness outcomes.

**Level of evidence:**

III.

## Background

Total knee arthroplasty (TKA) is the most successful procedure for end-stage bone-on-bone knee osteoarthritis [1]. Cemented TKA has been the gold standard for over 40 years with reproducible results, but the technology has remained essentially unchanged [2]. Cementless fixation, by contrast, is evolving rapidly [3–5]. Most recently, additive manufacturing and 3D printing have produced surfaces that closely mimic cancellous bone properties, potentially improving very long-term outcomes [5–8].

Current evidence suggests that in younger, high-functional-demand, and obese patients, cementless TKA may offer equivalent or superior early outcomes and a theoretical long-term survival advantage through biological fixation [9–12]. Beyond these specific subgroups, however, the evidence comparing the two fixation methods has its own limitations [13–15]. Registry-based analyses, while offering large sample sizes, introduce variability in surgical technique, incomplete capture of patient-reported outcomes, and aggregated data that cannot be disaggregated to explore the nuances of individual patient response [16, 17]. Higher-quality comparative evidence, derived largely from randomized controlled trials, is methodologically robust but has focused almost exclusively on the superiority or non-inferiority of mean clinical outcomes, generally reporting equivalence between cemented and cementless fixation [18, 34–36].

Therefore, we hypothesized that a 3D-printed full-titanium cementless TKA and the same-design cemented implant would not differ significantly in average joint awareness at 2 years, but would differ in the proportion of patients with the poorest outcomes, with no difference expected at the upper end of the distribution. If biological osseointegration improves load transfer at the bone-implant interface, its benefit should be greatest in patients for whom that interface limits joint perception and negligible in those who already achieve near-complete joint unawareness, in whom a ceiling effect would be expected. The aim of this study was to test this hypothesis by comparing the two fixation methods using the Forgotten Joint Score-12, the Oxford Knee Score, and the Patient Acceptable Symptom State (PASS), assessing the effect of fixation both at the mean and across the full range of patient-reported outcomes.

## Materials and methods

### Design and population

A prospective non-randomised two-cohort investigation was conducted on a consecutive series of primary unilateral TKAs performed at a single institution between January 2021 and July 2022. The study was conducted in accordance with the Declaration of Helsinki and received IRB approval (no. CEAVC22198). Informed consent was obtained from all participants. The study was reported according to the STROBE guidelines [19].

All patients who underwent primary TKA for end-stage bone-on-bone knee osteoarthritis were assessed for eligibility. Exclusion criteria were valgus deformity and bilateral surgery. A total of 109 consecutive TKAs were included: 55 cemented and 54 cementless.

### Cohort allocation

Allocation was not randomised and was determined by surgeon preference. The cementless design was preferentially selected for younger patients, while the cemented version was preferred for older patients. As a result of this allocation, the groups differed significantly in age (cementless 66.5 ± 7.6 vs. cemented 74.6 ± 6.6 years, *P* < 0.001), sex distribution (48% vs. 24% male, *P* = 0.008), and BMI (28.1 ± 4.7 vs. 30.5 ± 5.2, *P* = 0.010), whereas preoperative OKS and ASA classification showed no significant difference.

### Surgical technique and rehabilitation

All procedures were performed using the GKS Prime Flex CR device with anterior stabilized polyethylene (Permedica, Merate, Italy). The femoral component of the cemented version is made of cobalt-chromium alloy, while the cementless version is fully 3D-printed using selective laser melting of Ti6Al4V (Traser^®^), featuring a highly porous (70%) and randomly irregular bone-facing surface with 520-µm pores engineered to maximize osseointegration. Both versions share an identical articular geometry and a titanium-niobium nitride articulating surface coating. The tibial components are both titanium-made; the cementless version is 3D-printed with a short keel, four bullet-shaped porous pegs, and a modular polished 2.5-cm stem, while the cemented version has a classic keel with a modular plug.

All procedures were performed by a single high-volume fellowship-trained adult knee reconstruction surgeon. Spinal anesthesia was used with a midline skin incision and medial parapatellar arthrotomy. Personalised low-pressure tourniquets (100mmHg more than systolic pressure at skin incision limited to 250 mmHg) were applied. Intravenous tranexamic acid (1000 mg in 100 mL saline) was administered before anesthesia, at the end of the procedure, and 3 h later. Intravenous cefazolin (2 g) was administered preoperatively and at 8 and 16 h postoperatively. Extramedullary and intramedullary guides were used for tibial and distal femoral cuts, respectively. Surgical procedures were conducted according to the measured resection technique with mechanical alignment. The patella was not resurfaced.

In the cemented group, simplex bone cement (Stryker, Mahwah, NJ) was applied to both the femoral and tibial components as well as the tibial keel. No stem extensions were used. In the cementless group, bone cuts were performed using a 1.27-mm saw blade under saline irrigation to avoid thermal necrosis. Thromboembolism prophylaxis was with enoxaparin sodium 4000 IU from 12 h after surgery and then once daily for 20 days. Rehabilitation involving full weight-bearing, walking, training, and isometric exercises was begun a few hours postoperatively according to fast-track protocol. Follow-up visits were performed at 3 weeks, 6 weeks, 6 months, 12 months, and 24 months.

### Data collection

Baseline demographics were collected including age, sex, body mass index (BMI), and American Society of Anesthesiologists (ASA) classification. Intraoperative data, perioperative complications, and any revision procedures were recorded. The Oxford Knee Score (OKS) was recorded preoperatively and at 24 months [20]. The Forgotten Joint Score-12 (FJS-12) was calculated at the 24 months follow-up [21]. Time to functional satisfaction was recorded prospectively by asking patients at each follow-up visit when they considered their knee joint to have reached a near-normal status. Patients who had not reached functional satisfaction by the 24-month follow-up were recorded as not having achieved this endpoint. Radiographic assessment including anteroposterior and lateral weight-bearing views was performed at each follow-up visit to evaluate implant positioning, radiolucent lines, and signs of loosening.

### Study endpoints

The primary outcome was the FJS-12 at 24 months, analysed both at the mean and across its distribution. The principal secondary outcome was the proportion of patients achieving the PASS for the FJS-12, defined as a score ≥ 33.3 according to Singh et al. [22]. Further secondary outcomes were the OKS at 24 months and its change from baseline, the time to functional satisfaction, and complications and revision.

### Data analysis

Baseline characteristics were summarised as means ± standard deviations for continuous variables and as frequencies (percentages) for categorical variables. Between-group comparisons of baseline characteristics used independent-samples t-tests or Mann–Whitney U tests for continuous variables and chi-square or Fisher exact tests for categorical variables, as appropriate.

FJS-12 at 24 months was analysed by multivariable linear regression adjusted for age, sex, BMI, and preoperative OKS, and, as a sensitivity analysis, by linear regression weighted by propensity score overlap weights estimated from the same covariates (standardised mean differences with 0.1 threshold). To examine whether the effect of fixation differed across the outcome range, quantile regression was performed at the 10th, 25th, 50th, 75th, and 90th percentiles, adjusted for the same covariates, using the quantreg package; standard errors and p-values were obtained by the xy-paired bootstrap with 2000 resamples, which does not assume homoscedasticity and is appropriate for tail estimation in moderate samples. This analysis was pre-specified but exploratory in nature, and p-values were corrected for multiple comparisons across the five quantiles using the Benjamini–Hochberg correction. The proportion of patients achieving the PASS for the FJS-12 (≥ 33.3) was compared between groups using Fisher exact test, with the absolute risk difference and number needed to treat calculated with 95% confidence intervals; Firth penalized logistic regression was used for covariate adjustment. OKS at 24 months and the change in OKS from baseline were analysed using overlap-weighted linear regression. Time to functional satisfaction was analysed as a time-to-event outcome, with patients not yet satisfied at 24 months censored at that point. Groups were compared by the log-rank test, and the adjusted effect of fixation was estimated by Cox proportional hazards regression adjusted for the same covariates. All effect estimates are reported with 95% confidence intervals. Statistical significance was set at *P* < 0.05 (two-sided). Analyses were performed in R version 4.5.1 (R Foundation for Statistical Computing, Vienna, Austria).

### Power analysis

To quantify the resolution of the mean comparison, we reported the detectable difference. With 55 and 54 patients per group and a pooled standard deviation of 19.8 points, the study was able to detect a between-group difference of approximately 10.7 FJS-12 points at 80% power (α = 0.05, two-sided). The study was therefore able to exclude large mean differences but was not powered to establish formal equivalence at the minimal clinically important difference; the distributional analyses (quantile regression and PASS achievement) constituted the pre-specified focus of the investigation.

## Results

A total of 109 patients were included with complete 2-year follow-up and none lost. There were 54 patients in the cementless group and 55 in the cemented group. The groups differed significantly in age, sex, and BMI, reflecting the allocation strategy, while preoperative OKS, ASA classification, and operative side were comparable (Table 1).

**Table 1:** Baseline demographics and clinical outcomes

Variable	Cemented (*n* = 55)	Cementless (*n* = 54)	*P*
Age, years	74.6 ± 6.6	66.5 ± 7.6	< 0.001
Sex			0.008
Female	42 (76%)	28 (52%)	
Male	13 (24%)	26 (48%)	
BMI, kg/m²	30.5 ± 5.2	28.1 ± 4.7	0.01
ASA class			0.6
1	4 (7.3%)	5 (9.3%)	
2	49 (89%)	49 (91%)	
3	2 (3.6%)	0 (0%)	
Operative side (right)	26 (47%)	25 (46%)	> 0.9
Preoperative OKS	13.4 ± 6.2	14.6 ± 6.0	0.4
OKS at 24 months	42.3 ± 7.0	42.1 ± 5.0	0.2
FJS-12 at 24 months	57.4 ± 21.6	64.9 ± 17.7	0.084†
TFS, months, median (IQR)	4 (3–6)	5 (4–11)	0.022†
PASS achieved (FJS-12 ≥ 33.3)	42 (76.4%)	52 (96.3%)	0.004‡

Note: Continuous variables as mean ± SD unless stated. Categorical as n (%). †Mann–Whitney U test. ‡Fisher exact test. Other continuous comparisons by independent t-test, categorical by chi-square

### Joint awareness at the mean

Cementless fixation was not a significant predictor of mean FJS-12 at 24 months after adjustment for age, sex, BMI, and preoperative OKS (β = 7.4, 95% CI − 1.8 to 16.6, *P* = 0.115). Overlap weighting, which achieved exact balance of all covariates, produced a closely concordant estimate (β = 7.3, 95% CI − 1.1 to 15.7, *P* = 0.086), indicating that the observed demographic imbalance did not account for the result (Table 2). On average, therefore, no significant difference between the two fixation methods was detected, although the confidence interval did not exclude a clinically relevant difference.

**Table 2:** Effect of fixation on FJS-12 at 24 months demonstrated by multivariable linear regression (primary) and overlap-weighted regression (sensitivity)

Variable	Primary β (95% CI)	*P*	Overlap-weighted β (95% CI)	*P*
Fixation (cementless vs. cemented)	7.4 (− 1.8 to 16.6)	0.115	7.3 (− 1.1 to 15.7)	0.086
Age (per year)	0.05 (− 0.49 to 0.60)	0.851	0.34 (− 0.50 to 1.19)	0.418
Sex (male vs. female)	−4.0 (− 13.2 to 5.2)	0.388	−0.7 (− 10.5 to 9.0)	0.88
BMI (per unit)	−0.56 (− 1.4 to 0.24)	0.165	−0.58 (− 1.4 to 0.21)	0.149
Preoperative OKS (per point)	0.19 (− 0.48 to 0.87)	0.572	0.003 (− 0.68 to 0.68)	0.994

Notes: Positive β indicates higher (better) FJS-12 in the cementless group

### Joint awareness across the distribution

**Fig. 1:**
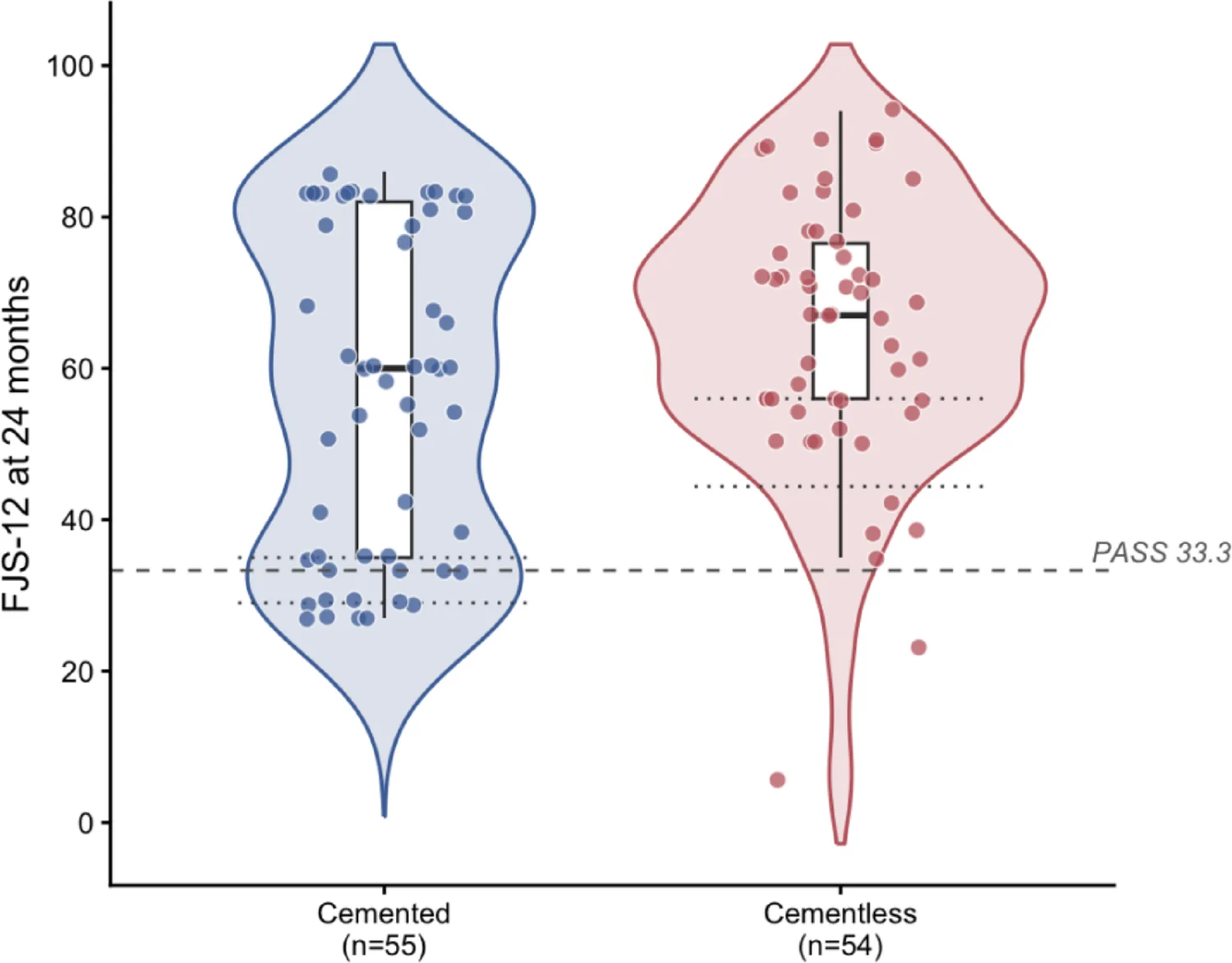
Distribution of the Forgotten Joint Score-12 (FJS-12) at 24 months by fixation type. Each point represents an individual patient (cemented, *n* = 55; cementless, *n* = 54), jittered horizontally to avoid overplotting. Violin shapes show the estimated probability density, and box plots show the median, interquartile range, and whiskers (1.5 × IQR). Dotted horizontal lines within each group mark the 10th and 25th percentiles. The dashed horizontal line indicates the Patient Acceptable Symptom State (PASS) threshold for the FJS-12 (33.3)

The effect of fixation was not uniform across the FJS-12 distribution (Table 3). Cementless patients scored substantially higher at the lower end, by 15.4 points at the 10th percentile and 17.4 points at the 25th percentile, with both differences remaining significant after correction (adjusted *P* = 0.028 and 0.021). No significant difference was detected at the median or upper percentiles. The advantage of cementless fixation was therefore concentrated among the patients at greatest risk of poor joint awareness, while those who already achieved high scores did so regardless of fixation (Fig. 1).

**Table 3:** Effect of fixation across the FJS-12 distribution demonstrated by quantile

Regression				
Quantile	β	SE	P	Adjusted P
10th	15.4	6	0.011	0.028
25th	17.4	6	0.004	0.021
50th	9.3	6.8	0.175	0.292
75th	−2.2	5.1	0.668	0.668
90th	4.6	4.2	0.285	0.357

Notes: β represents the adjusted difference in FJS-12 points attributable to cementless fixation at each quantile. Positive values indicate higher (better) scores in the cementless group. Adjusted for age, sex, BMI, and preoperative OKS

Consistent with this, a greater proportion of cementless than cemented patients achieved the PASS for the FJS-12 (96.3% vs. 76.4%), an absolute difference of 19.9% (95% CI 7.1 to 32.9), corresponding to a number needed to treat of 5 (95% CI 3 to 13). The difference remained significant after adjustment for age, sex, BMI, and preoperative OKS (Firth penalized logistic regression, *P* = 0.005); age in particular was not significantly associated with PASS achievement (*P* = 0.98), indicating that the higher rate in the cementless group was not explained by its younger age.

### Secondary outcomes

Fixation was not significantly associated with the OKS at 24 months (β = −0.2, 95% CI − 3.3 to 3.0, *P* = 0.91) or the change in OKS from baseline (β = −0.2, *P* = 0.93). Time to functional satisfaction was longer in the cementless group on unadjusted comparison (log-rank *P* = 0.04), but this difference disappeared after adjustment; fixation was not associated with the time to reach functional satisfaction (HR = 0.82, 95% CI 0.52 to 1.29, *P* = 0.39), indicating that the slower unadjusted recovery reflected the demographic profile of the cementless group rather than the implant itself (Table 4). Seven patients (6.4%) had not reached functional satisfaction by 24 months (5 cementless, 2 cemented).

**Table 4:** Secondary outcomes (adjusted analyses)

OKS at 24 months (overlap-weighted linear regression)			
Variable	β	95% CI	P
Fixation (cementless vs. cemented)	−0.2	−3.3 to 3.0	0.91
Age (per year)	0.04	−0.11 to 0.20	0.594
Sex (male vs. female)	−1.2	−4.9 to 2.6	0.538
BMI (per unit)	−0.14	−0.40 to 0.12	0.287
Preoperative OKS (per point)	−0.04	−0.32 to 0.24	0.765
Change in OKS from baseline (overlap-weighted linear regression, without preoperative OKS)			
Variable	β	95% CI	P
Fixation (cementless vs. cemented)	−0.2	−4.3 to 4.0	0.935
Age (per year)	0.2	−0.02 to 0.42	0.081
Sex (male vs. female)	−5.6	−11.1 to − 0.15	0.044
BMI (per unit)	−0.21	−0.59 to 0.18	0.285
Time to functional satisfaction (Cox proportional hazards regression)			
Variable	HR	95% CI	P
Fixation (cementless vs. cemented)	0.82	0.52 to 1.29	0.39
Age (per year)	1.02	0.99 to 1.05	0.188
Sex (male vs. female)	0.61	0.37 to 1.01	0.055
BMI (per unit)	1	0.96 to 1.04	0.903
Preoperative OKS (per point)	1.01	0.98 to 1.05	0.513

Notes: HR > 1 indicates faster attainment of functional satisfaction

### Complications and revision

Complications occurred in 4 cementless patients (7.4%: one suture-material reaction, one manipulation under anaesthesia (MUA), one joint effusion, and one lower-limb oedema) and 7 cemented patients (12.7%: two MUA and one further case of stiffness managed conservatively, two wound-related complications [suture reaction and superficial dehiscence treated with negative-pressure dressing], one lower-limb oedema, and one reactive cutaneous eruption). All resolved without implant failure, revision, or loosening.

## Discussion

The most important finding of this study was that cementless TKA did not produce higher mean joint awareness scores than the same-design cemented device, but it significantly reduced the proportion of patients who failed to achieve satisfactory joint awareness at 24 months. While no significant difference in mean FJS-12 was detected between groups, the cementless implant conferred a substantial benefit at the lower end of the outcome distribution. Moreover, a significantly greater proportion of cementless than cemented patients achieved the PASS for the FJS-12, a difference that remained significant after adjustment for age and other covariates. These findings suggest that the 3D-printed full-titanium cementless implant did not prove to be better on average, but more reliable, producing more consistent and predictable joint awareness outcomes with fewer subjective failures.

Patients in the lowest percentiles of joint awareness are those who persistently “feel” their prosthesis, experiencing residual discomfort, a sensation of foreign body, or mechanical awareness during daily activities. In these patients, the characteristics of the bone-implant interface may play a more prominent role [5–8]. Cementless fixation is engineered to achieve biological osseointegration that mimics the trabecular architecture and elastic modulus of native bone [23–26]. This may result in a more physiological load transfer at the interface compared with a rigid cement mantle, potentially reducing periarticular stress and the perception of a foreign body [6, 25, 27, 28]. Furthermore, the lower elastic modulus of titanium relative to cobalt-chromium may contribute to reduced stress shielding, which has been hypothesized to influence periprosthetic bone remodeling and patient comfort [29–32]. Conversely, the advantage of cementless fixation faded among patients with higher FJS-12 scores, likely reflecting a ceiling effect whereby joint awareness is already minimal and the contribution of implant-related factors becomes limited.

No significant difference in the speed of recovery was detected between the two fixation methods. Although the cementless group reached functional satisfaction later in the unadjusted comparison, the difference disappeared after adjustment for covariates, and fixation type had no independent effect on the speed of recovery. This was consistent with systematic evidence showing no difference in short-term pain or early function between cemented and cementless TKA [33].

Few studies have directly compared cemented and cementless TKA devices of the same design, and the available evidence derives from a small number of randomized controlled trials [18, 34–36].

Nam et al. [18] and Nivbrant et al. [36] found no difference in clinical outcomes at 2 years; at 5-year follow-up, Fricka et al. [34] reported equivalent clinical outcomes with the same implant system; and at 6 years, Hannon et al. [35] confirmed equivalent functional scores. All these studies relied on mean score comparisons, which may have obscured heterogeneous treatment effects across the outcome distribution. To our knowledge, the application of quantile regression to this comparison is novel in the cemented versus cementless literature.

This study demonstrated that cementless fixation can reduce the rate of poor subjective outcomes without improving mean scores. This distinction was not previously reported, while the evidence has focused almost exclusively on superiority or non-inferiority. The quantile regression allowed the identification of a heterogeneous treatment effect that would have been entirely missed by conventional mean-based comparisons and may serve as a methodological template for future studies investigating implant performance across the full spectrum of patient outcomes.

This study alone will not change clinical practice, nor should it. However, it generated a testable hypothesis that the benefit of modern cementless TKA may lie not in improving average outcomes, but in raising the floor of patient experience. Confirmation through adequately powered randomized trials or large registry analyses is warranted before these findings can inform clinical decision-making.

This study has several limitations. Allocation was by surgeon preference, directing the cementless implant toward younger patients; residual confounding from unmeasured determinants of joint awareness (activity, mental health, comorbidity, bone quality, expectation) cannot be excluded, and overlap weighting balances only measured covariates. The study was underpowered for smaller differences; the non-significant mean result therefore reflects an absence of a detected difference, not equivalence. Being open-label, the patient-reported endpoints were susceptible to an expectation effect, though patients were unaware of the implant and any such bias would act uniformly. The quantile regression was exploratory with extreme-percentile estimates based on few patients; the single-surgeon, single-centre design aids internal consistency but limits generalizability. Radiographs were obtained as a routine safety assessment to exclude loosening and osteolysis rather than as a study endpoint, and were not scored with a standardised radiolucent-line system by an independent blinded reader. One author discloses royalties from the manufacturer, but as both implants are made by the same manufacturer and differ only in fixation this creates no directional incentive.

## Conclusions

The 3D-printed full-titanium cementless TKA did not produce a significantly higher mean FJS-12 than the same-design cemented device at 24 months, but it significantly reduced the proportion of patients with poor joint awareness outcomes. The cementless implant conferred a substantial benefit at the lower end of the outcome distribution, while patients achieving high scores did so regardless of fixation type. The 3D-printed full-titanium cementless TKA did not prove to be a “better” implant on average, but a more reliable one.

## Data Availability

The dataset is available on request.
